# Evaluation of Mood Check-in Feature for Participation in Meditation Mobile App Users: Retrospective Longitudinal Analysis

**DOI:** 10.2196/27106

**Published:** 2021-04-23

**Authors:** Jennifer Huberty, Jeni Green, Megan Puzia, Chad Stecher

**Affiliations:** 1 College of Health Solutions Arizona State University Phoenix, AZ United States; 2 Behavioral Research and Analytics, LLC Salt Lake City, UT United States

**Keywords:** adherence, meditation, mindfulness, mood, smartphone application, app, engagement, mHealth, mental health, behavior

## Abstract

**Background:**

Mindfulness meditation smartphone apps may improve mental health but lack evidence-based behavioral strategies to encourage their regular use for attaining mental health benefits. In October 2019, the Calm mindfulness meditation app introduced a mood check-in feature, but its effects on participation in meditation have yet to be tested.

**Objective:**

The objective of this study was to investigate how a mood check-in feature impacts meditation behavior in Calm app subscribers.

**Methods:**

This was a retrospective longitudinal analysis of mobile app usage data from a random sample of first-time subscribers to the Calm app (n=2600) who joined in summer 2018 or summer 2019. The mood check-in feature allows users to rate their mood using an emoji after completing a meditation session and displays a monthly calendar of their past mood check-ins. Regression analyses were used to compare the rate of change in meditation behavior before and after the introduction of mood check-ins and to estimate how usage of mood check-ins was associated with individuals’ future meditation behavior (ie, intent-to-treat effects). Additional regression models examined the heterogenous effect of mood check-ins between subscribers who were active or inactive users prior to the introduction to mood check-ins (ie, above or below the median number of weeks with any meditation within their cohort). In order to confirm the specific associations between mood check-ins and meditation engagement, we modeled the direct relationship between the use of mood check-ins in previous weeks and subsequent meditation behavior (ie, treatment on the treated effects).

**Results:**

During the first 9 months of their subscription, the 2019 cohort completed an average of 0.482 more sessions per week (95% CI 0.309 to 0.655) than the 2018 cohort; however, across both cohorts, average weekly meditation declined (–0.033 sessions per week, 95% CI –0.035 to –0.031). Controlled for trends in meditation before mood check-ins and aggregate differences between the 2018 and 2019 samples, the time trend in the number of weekly meditation sessions increased by 0.045 sessions among the 2019 cohort after the introduction of mood check-ins (95% CI 0.039 to 0.052). This increase in meditation was most pronounced among the inactive subscribers (0.063 sessions, 95% CI 0.052 to 0.074). When controlled for past-week meditation, use of mood check-ins during the previous week was positively associated with the likelihood of meditating the following week (odds ratio 1.132, 95% CI 1.059 to 1.211); however, these associations were not sustained beyond 1 week.

**Conclusions:**

Using mood check-ins increases meditation participation in Calm app subscribers and may be especially beneficial for inactive subscribers. Mobile apps should consider incorporating mood check-ins to help better engage a wider range of users in app-based meditation, but more research is warranted.

## Introduction

There are an estimated 20,000 mental health smartphone apps currently available for download [1]. Mindfulness meditation apps are a popular type of mental health app, with over 290 available for download [1]. To date, Headspace and Calm are the leading mindfulness meditation apps with 65 and 100 million total downloads, respectively [2,3]. Interventions using mindfulness meditation apps indicate small- to medium-sized effects on improvements in depression and anxiety, life satisfaction, and positive affect [4], and greater engagement with mental health apps is associated with larger reductions in mental health symptoms [5]. Thus, mindfulness meditation apps are easily accessible, feasible, and cost-effective tools for promoting mental health and well-being on a large scale [4]. Despite the potential benefits of using these apps, not everyone remains engaged [5], and existing mobile Health strategies to increase mindfulness meditation app engagement have yet to identify techniques that can increase participation, both among subscribers who are active users (ie, currently using an app on a regular or semiregular basis) and those who are inactive users (ie, those who subscribed to an app but did not or no longer use it).

Lack of engagement is a well-documented barrier confronting mobile app interventions. A recent systematic review and meta-analysis [5] reported that participation in mental health app-based interventions consistently decreased over time, and percentages of participants who adhere to all prescribed sessions vary widely between studies and found that, in studies targeting depression, anxiety, or stress, average intervention adherence rates were 34%, 36%, and 41%, respectively. Adherence reported in studies [6,7] testing the Calm meditation app were similar. For example, in an 8-week intervention to improve stress in college students, only 22% of participants adhered to the prescribed meditation schedule (10 minutes per day) through the completion of the intervention [6]. In a study [7] to improve cancer-related symptoms (eg, fatigue, pain, anxiety, sleep disturbance) in patients with blood cancer, meditation participation decreased from an average of 86 minutes per week to 53 minutes per week over the course of the 4-week intervention period (a 38% decline). In both studies [6,7], adherence declined despite high initial motivation and high rates of satisfaction with the meditation app and perceived benefits of using apps to meditate. In real-world settings (eg, noninterventions), rates of app usage also decline rapidly; 71% of users stop using commercially available apps within 3 months of starting a subscription despite having sufficient initial interest and motivation to purchase an app subscription (which range from US $20 to $70) [8,9]. There is a limited understanding of how to re-engage meditation app subscribers after long periods of inactivity or after stopping app use entirely [10]. Identifying strategies to increase mobile app use among subscribers who are active or inactive is particularly important for developers of mindfulness meditation apps that are associated with large improvements in mental health only among subscribers who are highly active users [5].

Self-tracking app usage is a common component of existing mental health apps [11]. In general, apps that utilize behavioral self-tracking features have less usage reduction and a lower likelihood of abandonment compared to apps without these features [12]. Mood check-ins (ie, assessing and documenting one’s mood after meditation) may be a particularly useful self-tracking strategy to improve engagement in subscribers who are active users or re-engage subscribers who are inactive users of app-based meditation for several reasons. First, tracking one’s mood may help users better recognize the immediate benefits of meditation. Second, mood check-ins may increase emotional self-awareness, which may further improve mental health symptoms [11]. Third, mood check-ins align with several constructs from social cognitive theory, which suggests that mood check-ins may encourage behavior change by increasing positive reinforcement for meditation, self-efficacy, and self-regulation (ie, self-monitoring, judgment, affective reaction) [13,14]. Finally, as meditation requires continued practice in order to develop mindfulness skills and attain the corresponding mental health benefits, meditation may not always provide clear and immediate feedback [15]; therefore, mood check-ins may provide tangible feedback to the user regarding their mental health in the moment and over time. This feedback increases the salience of both the immediate and future benefits of meditation, and thus, may increase meditation engagement by combatting myopic loss aversion, a decision-making bias commonly noted by behavioral economists, in which short-term preferences lead people to undervalue health behaviors (eg, meditation) that produce future benefits [16]. Mood check-ins, therefore, have a strong theoretical justification for improving app engagement; however, their effect on active and inactive subscribers’ app-based meditation practices has not yet been evaluated.

In October 2019, the Calm mindfulness meditation app introduced a mood check-in feature that is based on constructs of social cognitive theory [13,14]. Therefore, the purpose of this study was to use app usage data from Calm subscribers to determine whether the introduction of mood check-ins within the Calm app increased meditation engagement.

## Methods

### Overview

We used an event-study design (or difference-in-differences model) to measure the change in meditation use after the introduction of mood check-ins among a random sample of users who had subscribed 3 months before mood check-ins were introduced, and compared that difference to the change in meditation use among a random sample of users who subscribed in the same month the year prior (ie, intent-to-treat effects). We also used this framework to examine whether mood check-ins had a heterogeneous effect on meditation engagement across subscribers in our sample who were active and inactive users prior to the introduction of mood check-ins (ie, above or below the median number of weeks with any meditation within their cohort). To confirm that it was specifically mood check-ins that increased engagement, we modeled the direct relationship between use of mood check-ins during the previous weeks and subsequent meditation behavior (ie, treatment on the treated effects). These findings may help to design future behavioral interventions for promoting mindfulness meditation app engagement and thus improving subscribers’ mental health outcomes.

### Ethics

This was a retrospective longitudinal analysis of mobile app usage data and was approved by the Arizona State University institutional review board (STUDY 00011292). Consent for use of data was provided by subscribers who agreed to the Calm privacy policy, which states that user information may be used for research and shared with third parties in anonymous or aggregate form.

### Participants and Data Source

Usage data were compiled from a random sample of 2600 first-time Calm app subscribers who joined in June or July 2018 (n=1300) or in June or July 2019 (n=1300). Subscribers were eligible for selection based only on the date of their initial paid subscription to the Calm app and were chosen using a randomization function in SQL. The Calm app introduced the mood check-in feature in October 2019; therefore, users who joined in 2018 had not been exposed to mood check-ins over the first 15 to 16 months of their subscription (ie, control group), whereas those who subscribed in 2019 were introduced to mood check-ins between 3 and 4 months after subscribing. No other major changes were introduced in the app during these time periods. Data for both groups were restricted to the first 9 months of app use to avoid analyzing usage patterns during the COVID-19 pandemic (February 2020) among the group that joined in 2019.

### The Calm App

The Calm app is a meditation app that is commercially available. Calm provides general guided meditation (eg, 10-minute Daily Calm, various individual and series meditations, and sleep-specific meditations) grounded in mindfulness-based stress reduction [17] and Vipassana meditation [18] and Sleep Stories, grounded in sensory immersion and present moment awareness. The Daily Calm meditations are 10 minutes in length and include topics that change daily targeted for both the beginner and advanced meditator (eg, karma, distraction, self-compassion). The individual and series meditations include 3- to 30-minute meditations with topics such as stress, self-care, and anxiety (and include meditations for beginners). The series meditations are designed to be practiced daily for 7 days and include topics such as “7 Days of Soothing Pain,” “7 Days of Managing Stress,” or “7 Days of Calming Anxiety.” The sleep-specific meditations are intended to sooth the body and mind into sleep through deep, progressive relaxation. Finally, the Sleep Stories are designed to help users fall asleep while listening to a variety of stories including fiction or nature-based.

Calm recently added a mood check-in feature which allows participants to rate their mood using an emoji after completing a meditation session; users then receive preset feedback messages related to their mood selection (eg, let negative thoughts be, let them go) and a monthly calendar of past mood check-ins that is displayed after checking-in. This feature is consistent with behavioral strategies (eg, reinforcement, self-monitoring [13,14]) that may help individuals participate in and maintain health behaviors.

### Statistical Analyses

#### Cohort Effects of the Introduction of the Mood Check-In Feature (Intent to Treat)

App usage data (number of meditation sessions completed, total minutes of meditation with the app, and likelihood of completing any meditation session) were aggregated by week over the first 9 months of users’ subscriptions. To estimate the overall effect of mood check-ins on meditation behavior over time, we used linear regression analyses. We compared the rate of change in meditation behavior measures in the 2019 cohort before and after the introduction of mood check-ins with those of the 2018 cohort during the corresponding time period in the previous year. We used a difference-in-differences approach, in which estimations of changes in meditation behavior were allowed to differ linearly for the periods before and after the mood check-in feature was introduced to determine the intent-to-treat effect. Outcomes were regressed on to a linear measure of weeks since starting to use the app, an indicator variable equal to 1 for the 2019 sample, and an indicator variable equal to 1 for the weeks after the mood check-in feature became available in 2019 (evaluated for the same week in both the 2018 and 2019 cohort), as well as all 2-way and 3-way interactions between the three variables. The model coefficient of interest was the estimated parameter for the change in weekly time trend after mood check-ins became available to the 2019 cohort—the variable *weeks×2019 cohort×mood check-ins*. All analyses were conducted using Stata software (version 16.1; StataCorp LLC).

To investigate whether the effects of mood check-ins were similar for active versus inactive users, we created separate models for active and inactive subscribers. Active and inactive status was defined by the number of weeks in which a subscriber completed at least 1 meditation session during the first 17 weeks of their subscription (corresponding to the period before mood check-ins were introduced in 2019). Users were classified as active subscribers if they were above the median number of weeks with any meditation within their cohort or as inactive subscribers if they were below the median number of meditation weeks threshold within their cohort. Thresholds were 2 and 3 weeks, respectively, for the 2018 and 2019 cohorts.

#### Association Between Using Mood Check-ins and Future Meditation Behavior (Treatment on the Treated)

To confirm the specific direct relationship between using mood check-ins and meditation engagement (treatment on the treated effect), we examined associations between an individual’s use of mood check-ins during the previous week and that person’s future meditation behavior by creating a second set of linear models regressing (1) the likelihood of any meditation during the week and (2) the weekly number of meditation sessions on the number of mood check-ins during the prior week (ie, a 1-unit lag). For these analyses, weekly app usage data were assessed for a 16-week period—8 weeks prior to and following the release of the mood check-in feature in October 2019. We created models for the entire 2019 cohort, active subscribers, and inactive subscribers. To control for differences in aggregate meditation behavior between subscribers who did and did not use the mood check-in feature, the models controlled for estimated meditation behavior during the prior week. To examine the duration of associations between using mood check-ins and future meditation behavior, we created models predicting subsequent weekly meditation using mood check-ins and meditation behavior measured over the previous 7 weeks.

## Results

### Introduction of the Mood Check-in Feature (Intent-to-Treat Effect)

Summary statistics for weekly meditation practices using the Calm app between the 2018 and 2019 cohorts show significantly greater meditation usage among those who joined in 2019 versus 2018 (Table 1). Compared to users who joined Calm in the summer of 2018, those who joined in the summer of 2019 were 0.122 percentage points more likely to meditate with the Calm app in a given week (*P<*.001), meditated for an average of 0.587 more sessions per week (*P<*.001), and meditated for approximately 7.761 more minutes per week (*P<*.001). Similar patterns were observed among the active and inactive subscribers (Table 1).

**Figure 1:**
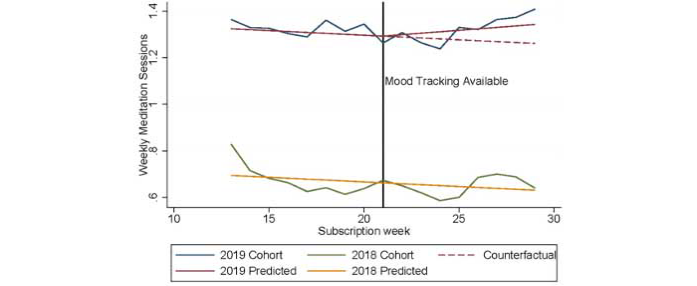
Average weekly meditation sessions for the 2018 and 2019 cohorts along with the estimated trend before and after the introduction of the mood check-in feature in October 2019.

The difference-in-differences model (Figure 1) shows that there were negative linear time trends in weekly meditation sessions occurring at an equal rate in both cohorts prior to the introduction of mood check-ins. This equivalence in trends prior to the introduction of the mood check-in feature for the 2019 cohort satisfies the parallel trends assumption (see Figure S1, Multimedia Appendix 1 for trends among active and inactive subscribers). An increase in meditation sessions occurred following the introduction of the mood check-in feature (Model 1, Table 2): across both cohorts, average weekly meditation sessions declined (–0.033 sessions per week, 95% CI –0.035 to –0.031), and in general, the 2019 cohort completed an average of 0.482 more sessions per week (95% CI 0.309 to 0.655) than the 2018 cohort completed. Importantly, the weekly change in meditation sessions significantly increased by 0.045 sessions per week (95% CI 0.039 to 0.052) in the 2019 cohort after the introduction of the mood check-in feature.

**Table 1:** Calm app usage between 2018 and 2019 cohorts during the full study period.

Subscriber meditation characteristics			2018 cohort^a^ (n=1300), mean (SD)		2019 cohort^b^ (n=1300), mean (SD)		Difference^c^		*P* value
**All subscribers^d^**									
	Any weekly meditation^e^	0.22 (0.42)		0.34 (0.48)		–0.122		*<*.001	
	Weekly meditation sessions	0.87 (2.49)		1.45 (3.28)		–0.587		*<*.001	
	Weekly meditation minutes	11.03 (35.54)		18.79 (46.14)		–7.761		*<*.001	
**Active subscribers^f^**									
	Any weekly meditation	0.39 (0.49)		0.57 (0.50)		–0.178		*<*.001	
	Weekly meditation sessions	1.56 (3.19)		2.51 (4.05)		–0.957		*<*.001	
	Weekly meditation minutes	19.83 (46.12)		32.48 (57.69)		–12.648		*<*.001	
**Inactive subscribers^g^**									
	Any weekly meditation	0.02 (0.14)		0.07 (0.26)		–0.052		*<*.001	
	Weekly meditation sessions	0.03 (0.30)		0.16 (0.94)		–0.129		*<*.001	
	Weekly meditation minutes	0.40 (3.77)		2.11 (12.72)		–1.708		*<*.001	

^a^Those who subscribed to the Calm app in June or July of 2018.

^b^Those who subscribed in either June or July of 2019.

^c^Difference between 2018 and 2019 cohort weekly averages.

^d^39,000 and 39,000 observations for the 2018 and 2019 cohorts, respectively.

^e^Any weekly meditation describes the estimated likelihood that a meditation session was completed during a given week.

^f^21,330 and 21,420 observations for the 2018 and 2019 cohorts, respectively.

^g^17,670 and 17,580 observations for the 2018 and 2019 cohorts, respectively.

Because the trends in weekly meditation sessions are not accurately described by a linear time trend over the study period, we focused our analyses on the 8-week periods before and after mood check-ins were introduced to the 2019 cohort and the same time period in the 2018 cohort (Model 2). The estimated effect of mood check-ins on weekly meditation sessions is attenuated but remains positive and statistically significant (Table 2). Specifically, the time trend in the number of weekly meditation sessions significantly increased by 0.012 over this period (95% CI 0.005 to 0.018). Based on this increased time trend, the cumulative effect of 8 weeks of mood check-ins was an estimated increase of approximately 1 meditation session. Similar time trends and statistically significant differences in app usage were observed when estimating the effect on weekly meditation minutes, resulting in an estimated increase of 5.1 minutes over the 8 weeks of mood check-ins (Table S1 and Figure S2-S3, Multimedia Appendix 1).

Table 2 shows that the primary effect of mood check-ins on meditation behavior was experienced by inactive subscribers. Specifically, the cumulative effect of 8 weeks of mood check-ins was an estimated increase of 1.7 weekly sessions for the inactive subscribers.

**Table 2:** Effect (ordinary least squares estimates) of the introduction of the mood check-in feature on average weekly meditation sessions.

Subscriber variables			Model 1: full study period				Model 2: 8 weeks before and after		
			Coefficient (95% CI)		*P* value		Coefficient (95% CI)		*P* value
**All subscribers^a^**									
	Subscription week	–0.033 (–0.035, –0.031)		<.001		–0.006 (–0.009, –0.003)		<.001	
	2019 cohort	0.482 (0.309, 0.655)		<.001		0.623 (0.455, 0.791)		<.001	
	Week×2019 cohort×mood check-ins	0.045 (0.039, 0.052)		<.001		0.012 (0.005, 0.018)		<.001	
**Active subscribers^b^**									
	Subscription week	–0.060 (–0.063, –0.056)		<.001		–0.014 (–0.019, –0.008)		<.001	
	2019 cohort	0.811 (0.528, 1.094)		<.001		1.061 (0.781, 1.341)		<.001	
	Week×2019 cohort×mood check-ins	0.063 (0.052, 0.074)		<.001		0.004 (–0.007, 0.015)		.46	
**Inactive subscribers^c^**									
	Subscription week	–0.001 (–0.002, 0.000)		.20		0.003 (0.001, 0.005)		.003	
	2019 cohort	0.070 (0.034, 0.107)		<.001		0.082 (0.026, 0.138)		.004	
	Week×2019 cohort×mood check-ins	0.026 (0.023, 0.029)		<.001		0.021 (0.017, 0.026)		<.001	

^a^78,000 and 49,400 observations for model 1 and model 2, respectively.

^b^42,750 and 27,075 observations for model 1 and model 2, respectively.

^c^32,250 and 22,325 observations for model 1 and model 2, respectively.

### Association Between Using Mood Check-Ins and Future Meditation Behavior (Treatment on the Treated)

Table 3 presents the average weekly meditation behavior between those among the 2019 cohort who did and did not use the mood check-in feature. Compared to nonusers, subscribers who used the feature were more likely to meditate, completed more meditation sessions, and spent more time meditating in a given week.

**Table 3:** Meditation behavior of users of mood check-ins vs nonusers of mood check-ins.

Subscriber meditation characteristics			Nonusers, mean (SD)		Users, mean (SD)		Difference^a^		*P* value
**All subscribers^b^**									
	Likelihood of any weekly meditation^c^	0.21 (0.40)		0.37 (0.48)		–0.122		*<*.001	
	Weekly meditation sessions	0.75 (2.93)		1.66 (3.25)		–0.587		*<*.001	
	Weekly meditation minutes	10.37 (44.48)		20.91 (44.85)		–7.761		*<*.001	
**Active subscribers^d^**									
	Likelihood of any weekly meditation	0.42 (0.49)		0.52 (0.50)		–0.178		*<*.001	
	Weekly meditation sessions	1.55 (4.04)		2.45 (3.78)		–0.957		*<*.001	
	Weekly meditation minutes	21.31 (62.48)		30.97 (52.94)		–12.648		*<*.001	
**Inactive subscribers^e^**									
	Likelihood of any weekly meditation	0.05 (0.22)		0.13 (0.34)		–0.052		*<*.001	
	Weekly meditation sessions	0.16 (1.48)		0.36 (1.32)		–0.129		*<*.001	
	Weekly meditation minutes	2.43 (20.78)		4.41 (16.67)		–1.708		*<*.001	

^a^Difference between nonuser and user weekly averages.

^b^4660 and 8340 observations for nonusers and users, respectively.

^c^Any weekly meditation describes the likelihood that a meditation session was completed during a given week.

^d^1960 and 5180 observations for nonusers and users, respectively.

^e^2700 and 3160 observations for nonusers and users, respectively.

To examine the association between using mood check-ins and future meditation behavior, we estimated the relationship between using mood check-ins during the prior week and the likelihood of any meditation during the subsequent week for the 8 weeks after mood check-ins were introduced. The results are presented in Table 4. All models control for the aggregate trends in the likelihood of any weekly meditation both before and after the introduction of mood check-ins, as well as an indicator for any weekly meditation during the previous week. Using the mood check-ins in the previous week increased the odds of meditating by 1.132 (95% CI 1.059 to 1.211). This pattern was similar across active and inactive subscribers.

**Table 4:** Effect (logistic panel regression estimates) of past mood check-ins on the likelihood of weekly meditation.

Predictor	All Subscribers (n=75,400)		Active subscribers (n=41,325)		Inactive subscribers (n=34,075)	
	OR^a^ (95% CI)	*P* value	OR (95% CI)	*P* value	OR (95% CI)	*P* value
Subscription week	0.963 (0.959, 0.966)	<.001	0.957 (0.953, 0.961)	<.001	1.005 (0.995, 1.015)	.33
2019 cohort	2.286 (1.949, 2.681)	<.001	2.055 (1.813, 2.330)	<.001	3.269 (2.697, 3.963)	<.001
Week×2019 cohort×mood check-ins	1.041 (1.030, 1.053)	<.001	1.024 (1.012, 1.037)	<.001	1.047 (1.022, 1.073)	<.001
Lagged 1-week meditation minutes	1.040 (1.038, 1.041)	<.001	1.038 (1.037, 1.040)	<.001	1.043 (1.038, 1.047)	<.001
Lagged 1-week total mood check-ins	1.132 (1.059, 1.211)	<.001	1.127 (1.047, 1.213)	.001	1.147 (1.011, 1.301)	.03

^a^OR: odds ratio.

To examine the long-term relationship between past mood check-ins and subsequent meditation behavior, we estimated associations between 7 weeks of lagged mood check-ins and future meditation engagement (Table S3, Multimedia Appendix 1). Each model also controlled for aggregate trends in the likelihood of any weekly meditation among each cohort over the sample period. The results showed that any past meditation was significantly associated with greater odds of meditation in a given week, but importantly, the magnitude of this positive relationship declined for meditation that occurred further in the past. The predictive relationship between using the mood check-in feature and the odds of future meditation was not sustained beyond 1 week.

## Discussion

To our knowledge, this is the first study to examine if the introduction of a mood check-in feature within a mindfulness meditation app increased meditation engagement. Our findings demonstrate that users were more likely to participate in meditation after mood check-ins were available (ie, 0.045 more weekly sessions 2019 subscribers following the introduction of mood check-ins compared to 2018 subscribers during the corresponding time period) and that this effect was stronger for inactive subscribers than for active subscribers. Additionally, we found that use of mood check-ins specifically increased the likelihood of future meditation engagement, even when controlling for past meditation behavior and aggregate trends in meditation patterns over time. Use of mood check-ins during the previous week was positively associated with likelihood of meditating the following week; however, use of mood check-ins beyond 1 week prior was not predictive of future meditation.

The 2019 cohort had an estimated cumulative increase in meditation of approximately 1 session (or 5 minutes), 8 weeks after the introduction of mood check-ins, compared to that of the 2018 cohort. Additionally, subscribers who used mood check-ins (compared to those who did not use mood check-ins) were more likely to meditate, completed more meditation sessions, and spent more time meditating on a given week. Importantly, these associations were strongest among previously inactive users. These data highlight the potential for mood check-ins to help subscribers participate in meditation after a period of low engagement. Existing literature suggests mood self-tracking helps app users increase their awareness of their mood patterns and, as such, helps with self-regulation and emotional well-being [19]. The mood check-in feature was also designed to increase the salience of immediate meditation benefits. The mood check-in calendar display may help users better visualize the long-term benefits of meditation and perhaps maintain meditation behavior by improving self-efficacy or even acting as a behavioral reinforcement [14]. Additionally, the increased salience of future long-term mental health benefits may help users overcome present-biased time preferences, allowing users to better evaluate the merits of mindfulness meditation and thus increase their meditation practice. However, to the authors’ knowledge, no studies have disentangled the potential mechanisms through which mood check-ins impact the use of a consumer-based meditation app. Future research should further examine how meditation and health outcomes are impacted by mood check-ins and investigate the behavioral channels that underlie these effects. Additional studies are also needed to investigate the use of mood check-ins over longer periods of time to see if and how improvements in meditation habits are maintained.

The effect of mood check-ins was greater for inactive subscribers (ie, cumulative effect of 8 weeks of mood check-ins increased their meditation practice by an estimated 1.7 sessions) than it was for active subscribers (ie, practices did not change). To our knowledge, there is no research evaluating app-based meditation behaviors and factors that influence continued engagement. According to the Transtheoretical Model and the Stages of Change, there is a distinction between people in the precontemplation or contemplation stages and those in the action or maintenance stages [20]. Those in the action stage are actively involved in taking steps to practice a health behavior, while those in the maintenance stages have ongoing practice of a health behavior and are less likely to relapse [20]. In our data, inactive subscribers may be those in the precontemplation or contemplation stages and may benefit from reinforcement management (such as mood check-ins) to help them move into the action stage, while active subscribers may already be in the maintenance stage, working toward sustaining a behavior rather than increasing their efforts. Interventions using mobile apps may consider evaluating stages of change and incorporating specific reinforcement strategies, such as mood check-ins, to move participants from the action to the maintenance stage.

We were not able to determine whether the benefits of mood check-ins come from individuals consciously deciding to meditate based on their mood or if this effect occurs on a subconscious level. If occurring subconsciously, the meditation behavior may be a byproduct of improvements in mental health that are associated with better health behaviors [21-23]. Alternatively, mood check-ins may increase the perceived benefit of meditation, and thus increase performance of the behavior. Although testing the mechanisms of mood check-ins on meditation behavior was beyond the scope of this paper, this is an important area that warrants additional research. Future studies may benefit from deeper investigation into individuals’ experiences related to mood check-ins, such as the potential effects of positive and negative emotions, expectancies about mood regulation [24], self-efficacy, and use of self-feedback to alter thoughts, feelings, and behavior [25,26].

### Limitations

Although this is one of the first studies to assess the effects of mood check-in on participation in meditation, there are important limitations to be noted. First, we did not have any information about subscribers in the current sample beyond their subscription data and app usage patterns. Thus, we did not have information on potentially important covariates that may relate to app usage and meditation behavior and that may modify the effect of mood check-ins on meditation engagement. Future studies are encouraged to collect information on demographics (ie, gender, age, income, education, etc), mental health, and other theoretically important subscriber characteristics to assess the possible confounding impacts of these variables on the relationship between mood check-ins and meditation engagement. Second, we did not assess how users engaged with other behavioral strategies already embedded within the Calm app (ie, tracking time spent meditating, reminders, sharing meditation stats); therefore, it is unknown if mood check-ins operate alone or in tandem with these other features when promoting greater meditation behaviors. Future studies should evaluate the interaction of these behavioral strategies with mood check-ins and their combined effect on participation in app-based meditation. Also, this study analyzed changes in app usage during the first 9 months of users’ subscriptions with Calm, but these results may not be generalizable to potential changes in meditation behavior that would occur at other points in a user’s subscription. Finally, the cumulative effect of 8 weeks of mood check-ins was an increase of approximately 1 meditation session or 5 minutes of meditation. These findings were statistically significant (both *P*<.001), but it is unclear whether small increases in meditation over an extended period of time constitute meaningful changes associated with clinically significant health benefits. Given the dearth of literature related to meditation dosage, future research is warranted to determine the frequency and duration of meditation optimally needed to convey benefits to participants and whether effects of this magnitude relate to future behavior and health outcomes.

### Conclusions

Using mood check-ins increased meditation participation (in sessions or minutes) and the likelihood of meditation in general, having the greatest benefit for inactive meditators. Mobile apps aiming to encourage better health behaviors should incorporate evidence-based strategies such as mood check-ins to sustain behavior or increase meditation participation, but more research is warranted to examine mechanisms and confirm the findings presented herein. Mobile app developers may also utilize this information to better engage inactive subscribers in an effort to sustain their consumer base.

## Appendix

Multimedia Appendix 1 Results and figures from supplementary analyses.
