# Supplementary material for: Evaluation of Mood Check-in Feature for Participation in Meditation Mobile App Users: Retrospective Longitudinal Analysis
Source: JMIR Mhealth Uhealth. 2021 Apr 23;9(4):e27106. doi: 10.2196/27106 (PMC8105761; doi:10.2196/27106)
Supplement: Multimedia Appendix 1 [file mhealth_v9i4e27106_app1.docx]

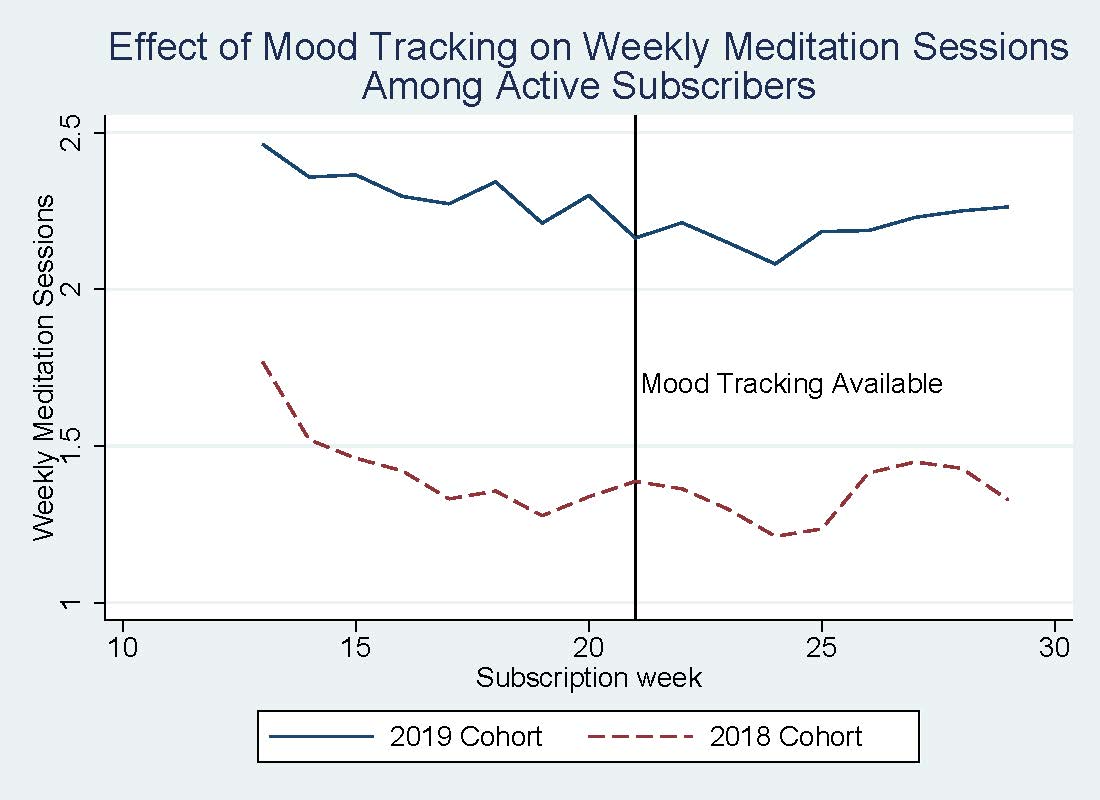


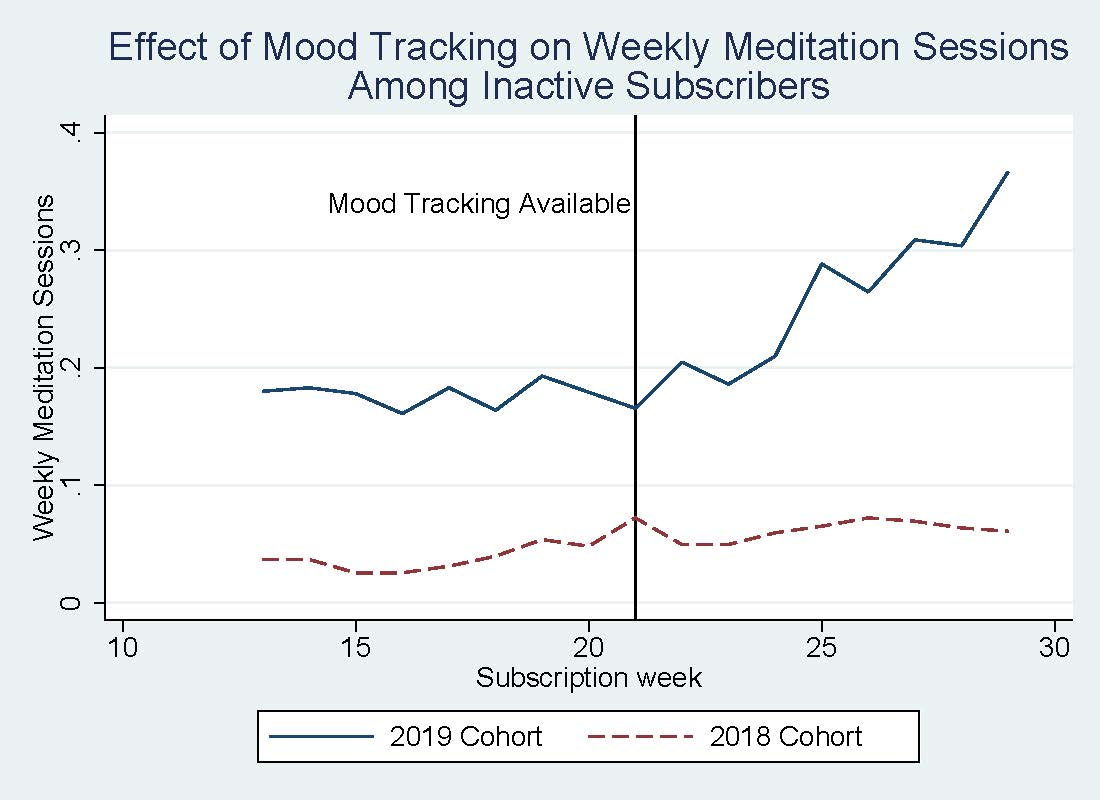


**Figure S1**: This figure displays the average weekly meditation minutes for the 2018 and 2019 cohorts before and after the introduction of mood check-ins in October 2019. When assessing app usage across the full duration of the study period, there were statistically significant increases in mediation engagement following the introduction of mood check-ins among both active subscribers (0.063^***^ [0.052, 0.074]) and inactive subscribers (0.026^***^ [0.023, 0.029]) compared to what would be expected based on the time trends in the 2018 cohort.

**Table S1. Average Weekly Meditation Minutes by Cohort**

|  | Model 1: | Model 2: |
| --- | --- | --- |
|  | Full duration of  The study period | +/- 8 wks. of  Mood Check-Ins |
| All subscribers |  |  |
| Subscription week | -0.418^***^ [-0.446, -0.391] | -0.085^**^ [-0.136, -0.034] |
| 2019 Cohort | 6.503^***^ [4.157, 8.848] | 8.206^***^ [5.881, 10.531] |
| Week x 2019 Cohort x Mood Check-Ins | 0.545^***^ [0.450, 0.640] | 0.143^**^ [0.041, 0.244] |
| Observations | 78,000 | 49,400 |

Note: This table presents the ordinary least squares (OLS) estimates of the difference-in-differences model of average weekly meditation minutes estimated for the full sample of observations from the start of joining Calm (Model 1) and for the eight-week period before and after mood check-ins were introduced to the 2019 cohort (Model 2). Subscription Week is a linear time trend, 2019 Cohort variable identifies users that joined in the summer of 2019, and Week x 2019 Cohort x Mood Check-Ins estimates a new linear time trend for the 2019 cohort after mood check-ins were introduced in October of 2019. 95% confidence intervals are presented in brackets; ^*^*P* < .05, ^**^*P* < .01, ^***^*P* < .001.


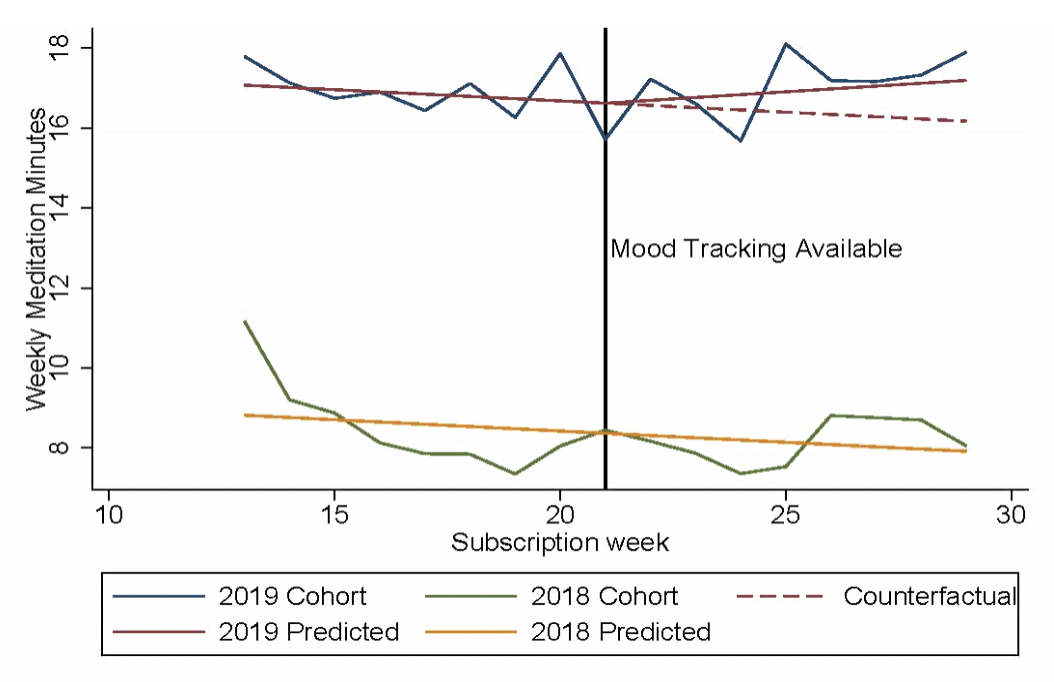


**Figure S2**: This figure displays the average weekly meditation minutes for the 2018 and 2019 cohorts along with the estimated trends in weekly meditation sessions before and after the introduction of mood check-ins in October 2019.


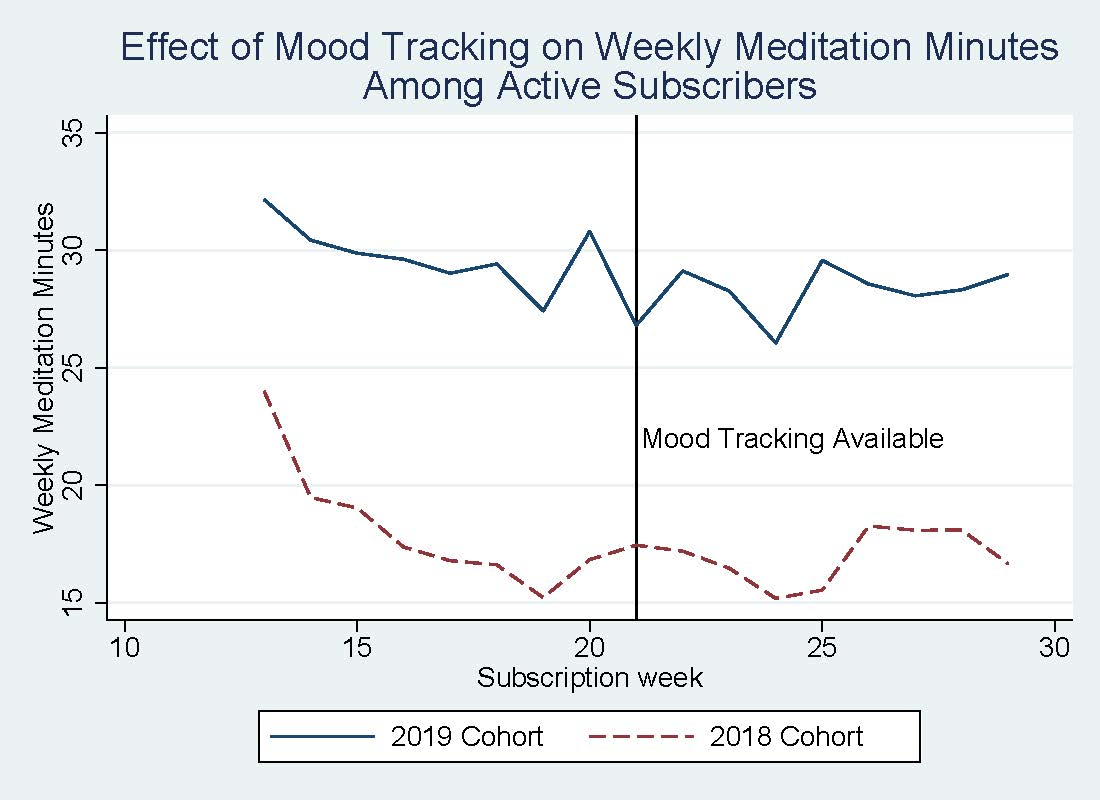

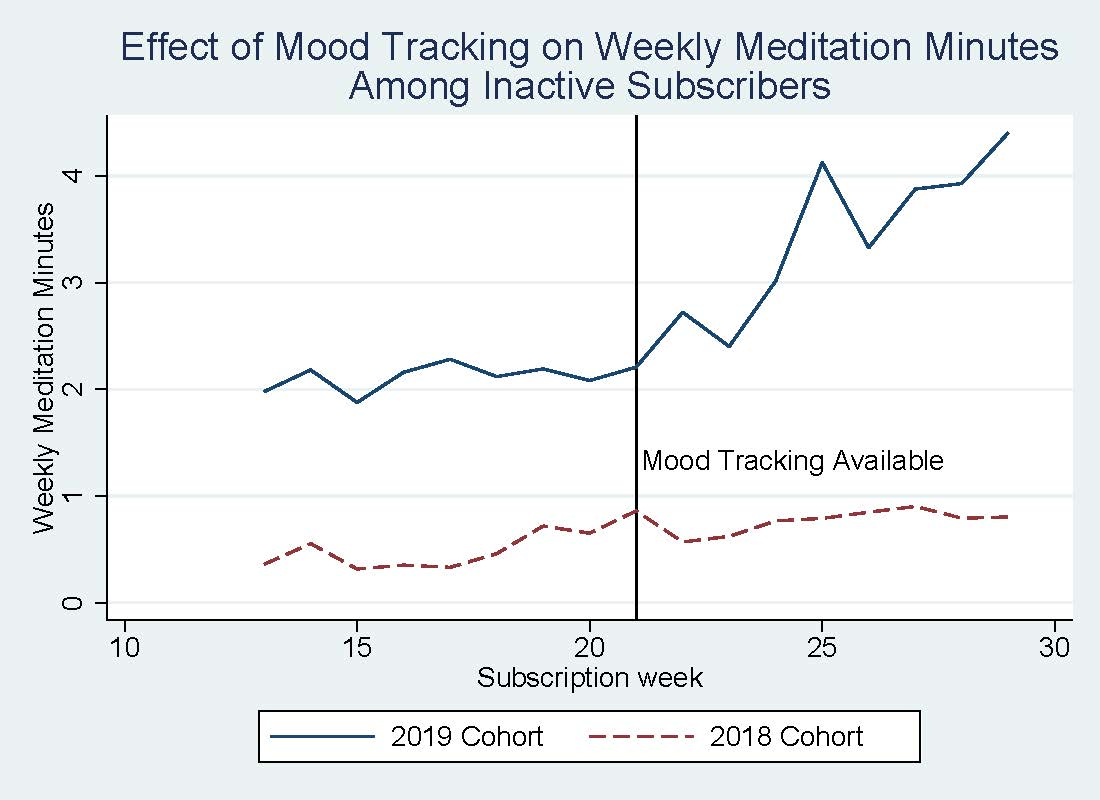


**Figure S3**: This figure displays the average weekly meditation minutes for the 2018 and 2019 cohorts before and after the introduction of mood check-ins in October 2019. When assessing app usage across the full duration of the study period, there were statistically significant increases in mediation engagement following the introduction of mood check-ins among both active subscribers (0.545^***^ [0.450, 0.640]) and inactive subscribers (0.143^**^ [0.041, 0.244] compared to what would be expected based on the time trends in the 2018 cohort.

**Table S2. Effect of Past Mood Check-ins on the Number of Weekly Meditation Sessions**

|  | Coefficient [95% CI] |
| --- | --- |
| All Subscribers |  |
| Subscription Week | -0.009^***^ [-0.011, -0.008] |
| 2019 Cohort | 0.302^***^ [0.230, 0.374] |
| Week x 2019 Cohort x Mood Tracking | 0.015^***^ [0.010, 0.020] |
| Lagged 1-Week Meditation Minutes | 0.030^***^ [0.029, 0.030] |
| Lagged 1-Week Total Mood Check-Ins | 0.059^***^ [0.031, 0.087] |
| Observations | 75,400 |
| Active Subscribers |  |
| Subscription Week | -0.019^***^ [-0.022, -0.016] |
| 2019 Cohort | 0.536^***^ [0.407, 0.665] |
| Week x 2019 Cohort x Mood Tracking | 0.016^***^ [0.007, 0.025] |
| Lagged 1-Week Meditation Minutes | 0.028^***^ [0.028, 0.029] |
| Lagged 1-Week Total Mood Check-Ins | 0.088^***^ [0.048, 0.129] |
| Observations | 41,325 |
| Inactive Subscribers |  |
| Subscription Week | 0.001^*^ [0.000, 0.002] |
| 2019 Cohort | 0.023^**^ [0.006, 0.039] |
| Week x 2019 Cohort x Mood Tracking | 0.013^***^ [0.010, 0.016] |
| Lagged 1-Week Meditation Minutes | 0.036^***^ [0.035, 0.037] |
| Lagged 1-Week Total Mood Check-Ins | -0.040^***^ [-0.061, -0.019] |
| Observations | 34,075 |

Note: This table presents the ordinary least squares (OLS) of the number weekly meditation on separate aggregate time trends in weekly meditation sessions between the 2018 and 2019 cohorts, as well as having number of meditation sessions in the prior week and using the mood check-ins feature in the prior week. 95% confidence intervals are in brackets; ^*^*p* < .05, ^**^*p* < .01, ^***^*p* < .001.

**Table S3. Likelihood of Any Meditation by Lagged Mood Check-Ins and Any Past Meditation**

| Predictor | Coefficient [95% CI] |
| --- | --- |
| Lagged 1-Week Any Meditation | 5.305^***^ [5.006, 5.622] |
| Lagged 2-Week Any Meditation | 2.594^***^ [2.437, 2.761] |
| Lagged 3-Week Any Meditation | 1.901^***^ [1.781, 2.028] |
| Lagged 4-Week Any Meditation | 1.612^***^ [1.508, 1.723] |
| Lagged 5-Week Any Meditation | 1.521^***^ [1.422, 1.628] |
| Lagged 6-Week Any Meditation | 1.531^***^ [1.432, 1.637] |
| Lagged 7-Week Any Meditation | 1.540^***^ [1.444, 1.643] |
| Lagged 1-Week Total Mood Check-Ins | 1.120^***^ [1.048, 1.196] |
| Lagged 2-Week Total Mood Check-Ins | 0.979 [0.909, 1.054] |
| Lagged 3-Week Total Mood Check-Ins | 0.957 [0.887, 1.033] |
| Lagged 4-Week Total Mood Check-Ins | 0.991 [0.914, 1.074] |
| Lagged 5-Week Total Mood Check-Ins | 1.025 [0.941, 1.116] |
| Lagged 6-Week Total Mood Check-Ins | 0.967 [0.883, 1.060] |
| Lagged 7-Week Total Mood Check-Ins | 1.051 [0.963, 1.147] |
| Observations | 59,800 |

Note: This table presents logistic regression estimates of the likelihood of any weekly meditation on separate aggregate time trends in weekly meditation between the 2018 and 2019 cohorts, as well as having any meditation in each of the prior seven weeks and using the mood check-ins feature in each of the prior seven weeks. The model controls for effects Subscription Week, 2019 Cohort, and the Week x 2019 Cohort x Mood Check-Ins interaction. Coefficients are presented as odds ratios and 95% confidence intervals are in brackets; ^*^ *P*< .05, ^**^*P* < .01, ^***^*P* < .001.
